# The use of individual cut points from treadmill walking to assess free-living moderate to vigorous physical activity in obese subjects by accelerometry: is it useful?

**DOI:** 10.1186/1471-2288-12-172

**Published:** 2012-11-15

**Authors:** Eivind Aadland, Jostein Steene-Johannessen

**Affiliations:** 1 Sogn og Fjordane University College, Faculty of Health Studies, Box 523, Førde, 6803, Norway; 2 Sogn og Fjordane University College, Faculty of Teacher Education and Sports, Box 133, Sogndal, 6851, Norway

**Keywords:** Exercise, Accelerometer, Actigraph, Individual calibration

## Abstract

**Background:**

Variation in counts between subjects at a given speed or work rate are the most important source of error in physical activity (PA) measurements with accelerometers. The aim of this study was to explore how the use of individual accelerometer cut points (ICPs) affected the analysis of PA field data.

**Methods:**

We performed a treadmill calibration protocol to determine cut points for moderate to vigorous PA (MVPA) (≥3 metabolic equivalents) and assessed free-living PA in 44 severely obese subjects using the Actigraph GT1M accelerometer. We obtained cut points in 42 subjects (11 men, mean (standard deviation) of body mass index (BMI) 39.8 (5.7), age 43.2 (9.2) years), of whom 35 had valid measurement of free-living PA (minutes of MVPA/day). Linear regression was used to analyze associations with the ICPs and time in MVPA/day. MVPA/day was also compared with values derived using a group cut point (GCP).

**Results:**

Resting oxygen consumption (partial r = 0.74, p < .001), work economy (partial r = −0.76, p < .001) and BMI (partial r = 0.52, p = .001) explained 68.4% of the variation in the ICPs (F = 26.7, p < .001). The ICPs explained 79.1% of the variation in the minutes spent in MVPA/day. Moderate to vigorous PA/day derived from the ICPs vs. the GCP varied substantially (R^2^ = 14%, p = .023, coefficient of variation = 45.1%).

**Conclusions:**

The results indicate that the use of ICPs had a strong influence on the PA level. Two thirds of the variation in the ICPs could be explained, however, a certain degree of measurement error will be present. Thus, we are not able to conclude with respect to the most appropriate procedure for analyzing time in MVPA.

## Background

Accelerometers have changed physical activity (PA) reporting from a self-reported estimate of intensity and duration to an objective measurement of bodily movement. Movements are quantified based on changes in accelerations and reported in the more or less arbitrary unit “counts”. To become meaningful, counts may be analyzed and interpreted in several different ways [1]. Because the health benefits of PA are determined, at least in part, by the work rate of the activity [2], the time spent at different work rates is one meaningful way to report the data. Thus, it is essential to establish accelerometer count cut points to separate sedentary behavior, light, moderate and vigorous PA, currently recommended to be defined as <1.5, 1.5-2.9, 3–5.9 and ≥6 metabolic equivalents (METs), respectively [2-4].

Numerous makes and models of accelerometers are currently in use to determine the PA level (e.g. Tritrac, Actigraph, BioTrainer, ActiTrac, Actical, Tracmor, Actiwatch, Sensewear, ActiHeart, ActiReg) [5-7]. In all cases, measurement variability of both technical and biological origin will be present. Accordingly, variability can be attributed to differences between devices (inter-instrument variation), differences over time (intra-instrument variation), differences between subjects (between-subject variation) and interactions between these different sources of variation. Currently, most methodological research concerning accelerometry has focused on calibrating (validating) accelerometer output according to some standard measure. However, because reliability is a premise for validity [8], research exploring measurement variability is important to reveal the different sources of variation (reviews of reliability studies for accelerometer measurement can be found elsewhere [5,9]). For instance, Actigraph instruments (Actigraph, Fort Walton Beach, FL, USA, formerly known as Computer Science and Applications (CSA) and Manufacture Technology Incorporated (MTI) models) are reported to have an inter-instrument coefficient of variation (CV) of under 8.9% [10-12] and an intra-instrument CV of under 4.4% [10,13]. The inter-instrument variation could be addressed through an individual unit calibration. However, a study by Moeller et al. [12] estimated that such a calibration explained under 4.2% of the variation in field data for Actigraph instruments. Hence, individual instrument calibration may not be worth the effort.

A greater source of variability is the variation in counts between subjects at a given speed or work rate. Two studies have applied a design allowing three-way analyses of variance between subjects, trials and instruments [11,14]. Both studies concluded that variation among subjects was by far the largest source of variability in the measurements. According to Barnett & Cerin [14], variation among subjects explained 89% of the total variation in the measured counts during a field walk. Welk et al. [11] reported that 63.4% of the variation in the counts during a treadmill walking trial could be ascribed to subject variation, whereas the remaining variation was explained to a greater or lesser extent by the interactions of trials and monitors with subjects. Hence, a calibration to individual subjects may be more important than the calibration of individual instruments. A procedure of this type is also recommended in the literature. This procedure is especially recommended for intervention studies because they require precise measurements at multiple time points [5,14,15].

We performed a treadmill calibration study of the Actigraph GT1M in 44 young to middle-aged severely obese subjects because cut points for use in this population is lacking and the equations for determining cut points may be population-specific [16]. The metabolic cost of walking increases with age and body weight [17-19], and this is not captured by an accelerometer [20,21]. The aim of the study was to explore how individual cut points (ICPs) for moderate to vigorous PA (MVPA) (≥3 METs) affected the assessment of free-living time spent in MVPA in severely obese subjects. We hypothesized that there would be no relationship between the ICPs and PA level.

## Methods

### Subjects

Forty-nine severely obese patients were enrolled at the Red Cross Haugland Rehabilitation Center in Norway between February 2010 and February 2011 to begin a lifestyle treatment program for obesity. The inclusion criteria for participation included an age between 18 and 60 years and a body mass index (BMI) > 40 kg/m^2^ without comorbidities, or a BMI > 35 with comorbidities. The exclusion criteria included pregnancy, heart disease, drug or alcohol abuse, previous bariatric surgery, and mental disorders and physical impairments that could reduce the subject’s ability to comply with the program. Written informed consent was obtained from each subject prior to inclusion in the study. This study met the standards of the Declaration of Helsinki and was approved by the Regional Committee for Medical Research Ethics.

### Procedures

#### Study protocol

The lifestyle treatment program was an intermittent in-patient program, and the first stay lasted six weeks. The PA level assessed by accelerometry was measured over a seven-day period about one month prior to the start of the lifestyle treatment program. Maximal oxygen consumption (VO_2max_) was measured in the first week, and the subjects who had little experience walking on a treadmill were advised to practice treadmill walking before the calibration study was performed. The calibration study was performed during the fourth week of the stay. The subjects visited the lab after a minimum of one hour of fasting and were not permitted to perform intense PA prior to the testing. They were weighed to the nearest 0.1 kg (BC 420 S MA, Tanita Corp, Tokyo, Japan) and were equipped with a heart rate monitor chest belt (Polar Electro Oy, Kempele, Finland) and an Actigraph GT1M accelerometer (Actigraph, Fort Walton Beach, FL, USA). Technical specifications of the accelerometer can be found elsewhere [22]. All the subjects wore an accelerometer attached in the mid axillary line of the right hip at the height of the umbilicus. Thirty different instruments were used in this case. In addition, 22 of the subjects wore an accelerometer at the left hip. In this case, the same instrument was used for every subject. The accelerometers were set at a 10-second epoch and a normal filtering option.

#### Treadmill calibration protocol and analysis

The test protocol consisted of two parts. First, the subjects were rested in a sitting position for 10 minutes to measure their resting oxygen consumption (resting VO_2_) according to the originally proposed definition of 1 MET [23]. Then, the subjects walked on the treadmill with no inclination for five minutes at 2, 3, 4, 5 and 6 km/h. Multiple treadmill speeds were checked manually to validate the treadmill speed. Oxygen consumption for the last seven minutes at rest and the last four minutes at each speed on the treadmill was measured using the Metamax I and the Metasoft v. 1.11.05 software (Cortex Biophysic, Leipzig, Germany). A one-point gas calibration using ambient air and a volume calibration using a three-liter syringe (SensorMedics Corporation, CA, USA) were performed between each test. The Metamax 1 analyzer has been shown to have no systematic error and a random error of 4% compared to the Douglas bag technique [24].

The last two minutes at rest and the last two minutes at each treadmill speed were used to calculate the oxygen consumption and accelerometer counts. Both measurements were originally reported for 10-second periods and were summed to determine the mean values of the oxygen consumption/min and counts/min. The counts/min was calculated from the vertical axis using the comma separated values (CSV)-files exported from the ActiLife v.5.3 software (Actigraph, Fort Walton Beach, FL, USA). The oxygen consumption from walking was divided by the oxygen consumption at rest to express the values for the metabolic cost of walking as individually adjusted MET-values.

#### Measurement and analysis of PA

Subjects were instructed to wear the accelerometer at all times, except during water activities (swimming, showering) or while sleeping. All files were analyzed using the ActiLife v. 5.3 software. A wear-time of ≥ 10 hours/day for ≥ five days was used as the criterion for a valid measure. Periods of ≥ 60 minutes (allowing for ≤ 2 minutes of non-zero counts) were defined as non-wear time [9,25]. The PA level was reported as minutes of MVPA/day and time in bouts of MVPA/day. The time spent in bouts of MVPA was defined as consecutive time in MVPA of ≥ 10 min in duration, allowing for ≤ 2 minutes drop below the cut point.

### Statistical analyses

The individual cut points were obtained from ordinary linear regression. Each dataset was checked with a scatterplot, and the Pearson (r) correlation and standard error of the estimate (SEE) was calculated. Despite a quadratic fit was indicated in some individuals, this was omitted because we believe a linear fit would be more robust on the individual level having only five observations. For two subjects, the MVPA cut points were estimated to have negative values. These cut points were replaced with cut point values of 100 counts/min.

To compare the cut points obtained from the right and the left hip, we used a Bland-Altman plot showing the differences between the hips (left hip – right hip) as a function of the mean value of the two variables [26]. Because the data were deemed to be homoscedastic, the standard error of the measurement (SEM) and the limits of agreement (LoA) were calculated according to Hopkins [27] (SEM = standard deviation (SD) of the differences / √2; LoA = SEM * √2 * 1.96). A one-sample Wilcoxon test was used to test the mean differences between the opposite hips. The Spearman correlation coefficient (ρ) and CV (SD/mean) was used to compare MVPA derived from ICPs and the GCP because the data were deemed to be heteroscedastic.

A linear regression model was used to explore the relationships between the ICPs (dependent variable) and age, sex, body mass index (BMI), height, resting VO_2_ (ml/kg/min) and work economy (VO_2_ (ml/kg/min) at three km/h (independent variables). A quadratic regression model (i.e., including the second order term of ICPs) was used to determine the relationship between the ICPs and minutes of MVPA/day and minutes in bouts of MVPA/day (inclusion of a third order term did not improve the models), whereas only the first order term was used in the model for total PA level (counts/min) (inclusion of the second order term did not improve the model). The effect of the ICPs versus potential confounding variables on PA level were determined by 1) including the same independent variables as above (age, sex, BMI, height, resting VO_2_ (ml/kg/min) and work economy (VO_2_ (ml/kg/min) at three km/h)) in the model having PA level as the dependent variable and 2) the same model were repeated with inclusion of the first and second order term of the ICPs. Effects are reported as partial correlations (partial r).

A group cut point (GCP) was derived using a linear mixed model regression based on restricted maximum likelihood estimation. Subjects were given a random intercept and treadmill speed was defined as a repeated measure using an autoregressive (AR1) covariance structure. No other random effects were included. This yielded a cut point of 685 counts/min based on the following model: METs = 2.5276 + 0.000690*counts/min (CI for intercept 2.2456 to 2.8096, F = 319.3, p <.001; CI for slope 0.000626 to 0.000753, F = 470.0, p < .001). Although we found a significant effect of the quadratic term counts/min^2^, this was omitted for the purpose of comparison with the ICPs. Residuals were normally distributed. Median differences between time in MVPA/day and time in bouts of MVPA/day analysed using the GCP and the ICPs were tested using the Wilcoxon’s signed rank test for dependent samples. The relationships between the measures were tested using the Spearman's ρ.

The main analyses were performed using SPSS v. 19.0 (SPSS Inc., Chicago, USA). P < 0.05 indicated significant differences.

## Results

A total of 49 subjects were recruited to the lifestyle intervention program. Of these, 44 subjects performed the treadmill calibration procedure. After two subjects were excluded owing to accelerometer malfunction, 42 subjects (11 men) had valid accelerometer calibration data. The characteristics of the subjects are shown in Table 1. A total of 40 subjects had a valid free-living PA accelerometer-measurement. Thirty-five subjects had both valid calibration data and free-living PA data. Consequently, the analysis of the ICPs applied to the field data and the comparison of the field data results using the GCP and the ICPs was based on 35 subjects. In addition, 22 subjects wore an accelerometer on the left hip. Accordingly, the comparative analysis of the accelerometer data from opposite hips was based on 22 subjects.

**Table 1:** The subject characteristics

	**Total sample**	**Men**	**Women**
N	42	11	31
Age	43.2 (9.2)	42.1 (8.5)	43.6 (9.5)
Height (cm)	172.2 (9.1)	182.3 (8.0)	168.6 (6.4)
Weight (kg)	118.2 (18.2)	127.1 (16.0)	115.1 (18.0)
BMI (kg/m^2^)	39.8 (5.7)	38.3 (4.9)	40.4 (6.0)
WC (cm)	127.6 (13.2)	131.8 (11.3)	126.1 (13.7)
VO_2max_ (l/min)	3.29 (0.66) (n = 32)	4.16 (0.60) (n = 8)	3.00 (0.37) (n = 24)
VO_2max_ (ml/kg/min)	27.61 (5.19) (n = 32)	32.30 (5.41) (n = 8)	26.05 (4.15) (n = 24)
PA level (counts/min)	167.6 (64.8) (n = 40)	146.4 (68.4) (n = 10)	174.7 (63.1) (n = 30)
ICP	1151 (685)	1335 (720)	1087 (673)
Resting VO_2_ (l/min)	0.36 (0.07)	0.42 (0.09)	0.34 (0.05)
Resting VO_2_ (ml/kg/min)	3.04 (0.40)	3.26 (0.43)	2.96 (0.36)
Walking speed at 3 METs (km/h)	2.60 (0.57)	3.00 (0.37)	2.47 (0.58)

Mean (SD). *BMI* = body mass index, *WC* = waist circumference, *VO*_*2max*_ = maximal oxygen consumption, PA level = physical activity level; ICP = individual cut point; Resting VO_2_ = resting oxygen consumption; METs = metabolic equivalents.

**Figure 1:**
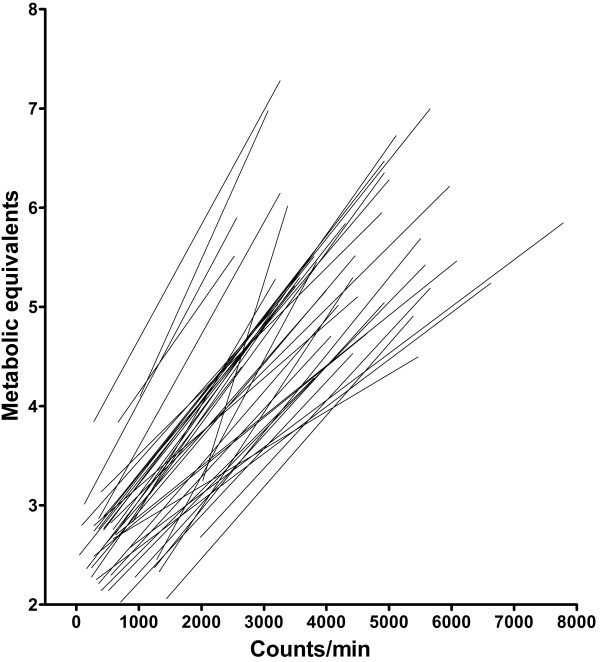
**Individual regression lines of counts/min vs. individual MET-values for 42 severely obese subjects.** Subjects walked on a treadmill at 2, 3, 4, 5 and 6 km/h.

The individual regression lines for the measurement at the right hip are shown in Figure 1 and mean (SD) of the ICPs for the group is shown in Table 1. The medians of the individual correlation coefficients between counts/min and the MET-values were r = 0.98 and r = 0.97 for the right and the left accelerometer, respectively. The corresponding SEEs were 0.29 and 0.34 METs.

**Figure 2:**
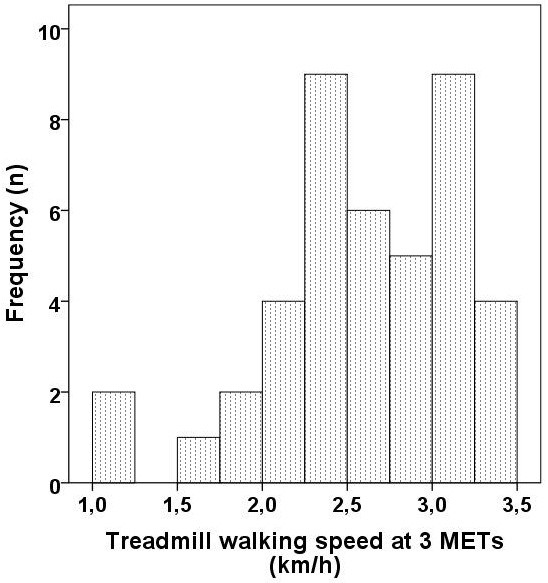
Histogram showing the distribution of treadmill walking speeds at three METs.

The walking speeds at three METs are shown in Figure 2. Minimum and maximum values were 1.1 and 3.5 km/h, respectively.

**Figure 3:**
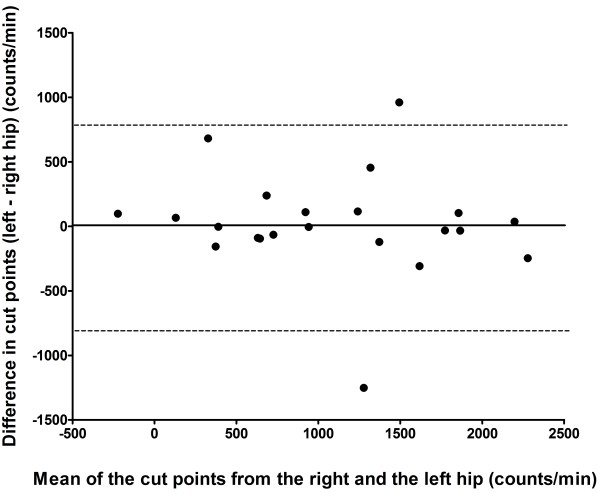
**Bland-Altman plot of cut points obtained from the right vs. the left hip.** Dotted lines are 95% limits of agreement.

Substantial differences were found between the counts/min measured at the right hip and at the left hip, with SEM and LoA values of 363 and 1007 counts/min, respectively. Figure 3 shows the Bland-Altman plot of the differences between the cut points calculated from the right and the left accelerometer. The mean ICP were similar between the hips (1078 (741) and 1095 (691) counts/min for the right hip and the left hip, respectively; difference (median, IQR) 7.4, 213.0 counts/min, p = .910). However, the individual differences were substantial, with a SEM of 288 counts/min and a LoA of 798 counts/min (95% LoA for the cut points at the left hip were −790.6 to +805.4 compared to the right hip).

Resting VO_2_ (partial r = 0.74, p < .001), work economy (partial r = −0.76, p < .001) and BMI (partial r = 0.52, p = .001) explained 68.4% of the variation in the ICPs (F = 26.7, p < .001). Resting VO_2_ (partial r = 0.98, p < .001) and work economy (partial r = −0.97, p < .001) explained 97.1% of the variation in treadmill walking speed at three METs (F = 647.0, p < .001).

**Figure 4:**
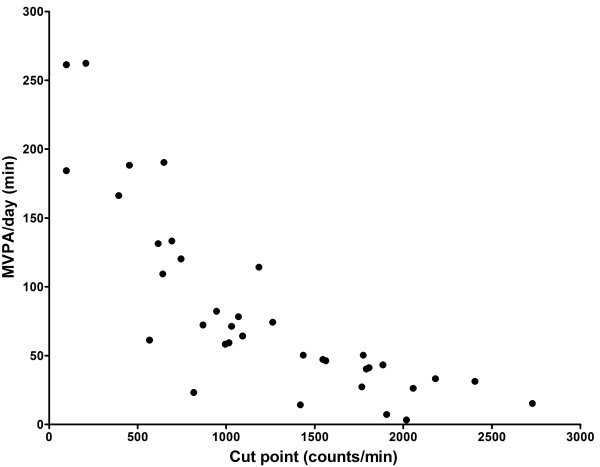
**Scatterplot showing the bivariate association between individual cut points and minutes of MVPA/day.** Quadratic R^2^ = 0.791 (p<.001).

Figure 4 shows the relationship between the ICPs and the minutes of MVPA/day. The ICPs alone explained 79.1% of the variance in the total minutes of MVPA/day (R^2^ = 0.791, F = 62.4, p < .001) and 62.5% of the variance in the minutes in bouts of MVPA/day (R^2^ = 0.625, F = 27.5, p < .001).

Resting VO_2_ (partial r = −0.50, p = .002), work economy (partial r = 0.55, p = .001) and age (partial r = 0.44, p = .009) explained 58.5% of the variation in the minutes of MVPA/day (F = 15.0, p < .001). When the ICPs were included in the model, only the first (partial r = −0.67, p < .001) and the second order term (partial r = 0.57, p < .001) of the ICPs were significant predictors for minutes of MVPA/day (resting VO_2_, work economy and age: partial r = 0.00 to 0.19, p = .296 to .983) (R^2^ = 0.803, F = 24.4, p < .001 for model). The results were very similar when bouts of MVPA were used as the dependent variable: Resting VO_2_ (partial r = −0.42, p = .013), work economy (partial r = 0.45, p = .008) and age (partial r = 0.49, p = .004) explained 53.5% of the variation (F = 12.3, p < .001), while only the ICPs were significant predictors when included in the model (resting VO_2_, work economy and age: partial r = −0.05 to 0.32, p = .073 to .803) (R^2^ = 0.668, F = 12.0, p < .001 for model). The ICPs was not related to total PA level (counts/min) in the bivariate model (R^2^ < 0.01, F = 0.1, p = .758) or in the multivariate model (partial r = 0.09, p = .636), nor was any other of the six independent variables used (partial r = −0.23 to 0.18, p = .228 to .983) (R^2^ = 0.13, F = 0.8, p = .783 for model).

On a group level, applying ICPs or a GCP yielded similar results for time in MVPA/day ((median (IQR) 60.1 (84.0) vs. 86.5 (35.3) min, respectively, p = .107) and bouts of MVPA/day (21.5 (45) vs. 29.7 (29.3) min, respectively, p = .272). The relationships between the measures were low to moderate (time in MVPA/day: ρ = 0.38, p = .023, CV = 45.1%; time in bouts of MVPA/day: ρ = 0.58, p < .001, CV = 51.2%).

## Discussion

The principal finding of this study was that the use of ICPs to determine the minutes of MVPA/day measured by accelerometry influenced the PA output substantially. Thus, minutes of MVPA determined by ICPs and a GCP varied considerably, indicated by low to moderate correlations and CVs of about 50% between the measures. This means that the use of ICPs will lead to quite different results on an individual level compared to using a common cut point on a group level. However, it is difficult to conclude with respect to the most appropriate procedure for analyzing time in MVPA.

The finding of a strong association between the ICPs and the measured minutes of MVPA/day was unexpected, as this indicates that subjects having lower ICPs were more physically active than subjects having higher ICPs. However, as we showed that two thirds of the variation in ICPs are due to resting VO_2_, work economy and BMI, most of the variation can be claimed to be true (although measurement error will be present). Since ICPs also attenuated the effects of all other independent variables used to explain the minutes of MVPA, one can argue that we managed to successfully determine ICPs that accounted for sources of variation that influenced work rate and thereby PA level. Thus, we can conclude that the use of ICPs in the present study increased the precision of the PA measurement. However, we will point out two main challenges when drawing this conclusion. First, one third of the variation in the ICPs was unexplained. Differences in gait patterns (beyond work economy), accelerometer-units worn, the attachment and tilt of the instruments are factors likely to explain this variation. The finding of a relatively large SEM/LoA for cut points obtained from the right and left hips, may support this notion (although these differences also could be attributed to biomechanical variations between the dominant and non-dominant side of the body). The attachment of the accelerometer may be a crucial aspect of the precision of the measurement process because tilting of the accelerometer or small differences in hip-placement are known to influence the accelerometer output [10,28]. We expected that the cut points derived from two accelerometers would be fairly similar and that typical deviations would be under 8.9% [10-12]. Because our data were homoscedastic, such a percentage difference is difficult to evaluate. However, the SEM amounted to approximately 27% of the group mean cut point. This result shows that the uncertainty in defining ICPs is substantial and that the percentage variation is greatest at the lower end. Further, because a regression toward the mean effect tended to be supported by the study data (i.e. subjects having high or low cut points from the right accelerometer had less extreme cut points from the left accelerometer), the established ICPs probably shouldn’t be viewed as “true” ICPs. It should be mentioned that the placement of the accelerometers probably would be less standardized in a field setting, compared to our laboratory setting, which would introduce even greater errors in the PA field data [11]. This unexplained variation will also be carried forward and influence the analyses of PA level. Our results revealed that use of ICPs to explain minutes of MVPA increased the explained variance with 10 – 20%, compared to the use of resting VO_2_, work economy and age as independent variables. This could imply that a certain degree of measurement error influence the results. Furthermore, measurement error in determining VO_2_ at rest and treadmill walking will add to this measurement error. Finally, any deviations in gait pattern and work economy between the treadmill walking and field will disturb the findings.

Second, if we believe that individual calibrations increase the measurement precision, we have to accept that use of a GCP may be more or less useless to determine minutes of MVPA on an *individual level*, as typical deviations is about 50% and shared variance is only 14% for total minutes of MVPA/day derived from ICPs vs. GCP. These differences are caused by great diversity in treadmill walking speed at three METs, which is accounted for by applying the ICPs. However, for describing PA level on a *group level*, applying ICPs or a GCP may lead to quite similar results, although we recognize that the PA levels derived from the GCP were about 40% higher than the PA level derived from the ICPs (because the estimated cut point from the regression model were somewhat lower than the mean of the ICPs) and that our study may suffer from lack of statistical power to detect a significant difference.

The present results suggest that severely obese individuals achieve a moderate work rate at very different walking speeds (between 1.1 and 3.5 km/h). Thus, because all movement detected above each individual’s walking speed threshold is interpreted as MVPA, it is not surprising that we found a strong association between ICPs and PA level. As stated above, the use of ICPs may significantly alter research findings compared to applying a GCP. Here we will consider three relevant areas. First, an interesting discussion is whether the use of ICPs can increase our ability to detect relationships between PA and diverse health outcomes. However, at this point we have no evidence-based suggestion for how different outcomes would be affected. Nevertheless, the difference between ICPs and GCP would probably be greater in cross-sectional analysis compared to experimental studies were the same cut point is applied repeatedly on an intra-individual level. Second, the use of ICPs vs. a GCP could influence whether subjects are found to achieve PA guidelines or not. In the present study we found a significant degree of re-classification between application of ICPs vs. the GCP (70% agreement, Kappa coefficient = 0.40, p = .299, result not shown) in the analyses of whether subjects achieved ≥ 30 min in bouts of > MVPA/day or not. Third, work rate can be determined absolutely (oxygen consumption or standardized MET-values etc.) or relative to an individual’s maximal work capacity (percentage of VO_2max_, etc.). This may challenge exercise prescription for subjects having low fitness levels, as “moderate” intensity exercise in absolute terms, can actually be quite demanding [29]. We could hypothesize that if resting metabolic rate, work economy and/or BMI (which determined the ICPs) was related to VO_2max_, applying ICPs could possibly relate better to each individual’s maximal capacity than a common GCP. However, we did not find any statistically significant relationship between ICPs and VO_2max_ (ml/kg/min) (r = 0.17, p = .320, result not shown).

### Strengths and weaknesses

The main strength of the present study is the use of precise and sophisticated measurements of the metabolic cost of walking and free-living PA. Moreover, the study gives an overview of the measurement properties of accelerometers and the use of individual cut points. Finally, the inclusion of a relatively large sample of subjects ensures the validity of our results.

This study has several limitations. The main limitation is that we do not have a valid criterion measure of minutes of MVPA/day, meaning that a direct comparison of the precision of a GCP vs. the ICPs could not be established. Because there is no “gold standard” for the measurement of time in various PA intensities, the criterion validity for the accelerometer measurements is impossible to establish [30,31]. Although PA measurements by accelerometry are found to be moderately correlated with measurements made using doubly labeled water [32], this technique is not suitable to measure minutes of PA at different work rates, as doubly labeled water only measure the total energy expenditure over a given time period [30,31]. Therefore, it is very difficult to perform a valid comparison of precision between the GCP and the ICPs. However, we established the relative validity using the short-form of the International Physical Activity Questionnaire (IPAQ) [33] (results not shown). The minutes spent in bouts of MVPA/day were moderately correlated with the IPAQ for both the GCP (Spearman’s ρ = 0.64, p = .001) and the ICPs (ρ = 0.51, p = .014) (n = 23). Because neither measure showed to be superior in comparison with the other, the finding does not encourage the use of one measure over another. However, it is very well known that objective and subjective PA outcomes are only moderately associated and that large variation are found between studies (mean correlation r = 0.37 (minimum r = −0.71 and maximum r = 0.98) based on 148 studies) [34]. Therefore, we believe our approach to the evaluation of applying ICPs should be viewed as a meaningful way to answer the research question asked. 

Second, our results may not be valid in a normal-weight population. As observed in the laboratory, the attachment of the accelerometers can be more challenging for severely obese subjects than for normal-weight subjects, and tilting of the instrument is known to reduce the level of counts [10]. In addition, musculoskeletal disorders and other factors that might interfere with walking capacity and work economy is much more common in severely obese subjects, compared to less obese and normal-weight subjects [35]. These effects may have produced greater variability in this population than would have been found in other populations. Thus, further research should verify or falsify our findings in a sample of less obese subjects.

Third, some issues regarding the performance of the calibration protocol and calculation of the ICPs deserve a comment. First, although we started the calibration procedure at a low speed to account for the high metabolic cost of walking in this group of severely obese subjects, 14 subjects spend more than three METs at two km/h. The extrapolation of the accelerometer counts to three METs may have caused some uncertainty in the count thresholds in these subjects. However, most subjects spent close to three METs at two km/h (n = 7 < 3.20; n = 11 < 3.50 METs). In addition, despite a quadratic fit between counts and metabolic cost was indicated in some individuals, we used linear models to derive ICPs to avoid overfitting of the models based on only five observations. This could have caused some uncertainty. However, applying ICPs derived from quadratic models did not change any findings. Second, we calibrated the accelerometers using a treadmill protocol, while PA was measured in a field setting. Although it doesn’t seem to be any agreement on which setting that causes the highest cut points (treadmill vs. track) [36,37], this may have caused variability on an individual level. Third, the participants started a lifestyle treatment program for their obesity in the period between performing the field measurement and the calibration protocol (i.e. one month prior to performing the calibration). This delay could have added variability to the results, as changes in physical fitness, resting metabolic rate or work efficiency could influence the relationship between accelerometer counts and work rate. Forth, the scaling of work rate to each individual subject’s resting metabolic rate to obtain individual MET-values, may have introduced a systematic bias relating to body size, as body composition is the most important determinant of the resting metabolic rate [38]. However, reanalysis of the data using a standardized MET value (3.5 ml/kg/min) as the reference for the calculation of work rate did not change any main findings. Fifth, as minutes of MVPA could be expected to increase with increased wear time, wear time could influence the findings. However, minutes of MVPA and percentage time in MVPA were very highly correlated (r = 0.98) and the use of percentage time in MVPA did not change any findings.

## Conclusions

We have shown that individual calibration of accelerometers based on a simple linear standard walking calibration approach performed at 2, 3, 4, 5 and 6 km/h influence the minutes of free-living MVPA to a great extent. Furthermore, we observed great diversity in time spent in MVPA when comparing the use of ICPs and a GCP to analyze PA level. It could therefore be argued that applying ICPs increase the measurement precision, but it is still difficult to draw a final conclusion. Moreover, the impact of using ICPs would probably depend on the research question being posed. If one should interpret with a conclusion that ICPs increase the measurement precision, one must also accept that a GCP applied on individual data are more or less useless to separate light PA from moderate-to-vigorous PA. We believe that there is an urgent need for further research that should explore the effect of applying ICPs versus a GCP to measure minutes of MVPA in less obese populations, as the use of ICPs may have the potential to increase measurement precision.

## Abbrevations

BMI: Body mass index; CV: Coefficient of variation; GCP: Group-level cut point; ICPs: Individual cut points; IPAQ: International physical activity questionnaire; IQR: Interquartile range; LoA: Limits of agreement; MET: Metabolic equivalent; MVPA: Moderate to vigorous physical activity; PA: Physical activity; Resting VO_2_: Resting oxygen consumption; SD: Standard deviation; SEE: Standard error of the estimate; SEM: Standard error of the measurement; VO_2max_: Maximal oxygen consumption.

## Competing interests

The authors declare that they have no competing interest.

## Author’ contributions

EAa participated in the design of the study, performed the physical testing, carried out the statistical analysis and drafted the manuscript. JSJ participated in the design of the study, the interpretation of the results and helped to draft the manuscript. Both authors read and approved the final manuscript.

## Pre-publication history

The pre-publication history for this paper can be accessed here:

http://www.biomedcentral.com/1471-2288/12/172/prepub
